# Modification of EDC method for increased labeling efficiency and characterization of low-content protein in gum acacia using asymmetrical flow field-flow fractionation coupled with multiple detectors

**DOI:** 10.1007/s00216-021-03587-y

**Published:** 2021-08-20

**Authors:** Meiyu Zhang, Lars Nilsson, Seungho Lee, Jaeyeong Choi

**Affiliations:** 1 Department of Chemistry, Hannam University, Daejeon, 34054 South Korea; 2 Department of Food Technology, Engineering and Nutrition, LTH, Lund University, 221 00 Lund, Sweden

**Keywords:** EDC method, Fluorescence-labeling, Low-content proteinaceous matters, Asymmetrical flow field-flow fractionation (AF4), Gum acacia (GA)

## Abstract

1-Ethyl-3-(3-dimethylaminopropyl) carbodiimide (EDC) is widely used as a crosslinker for fluorescence labeling of protein in the fields of biochemistry and food analysis. Many natural polysaccharides often contain some proteins or peptides that are very low in content but play a vital role in their biological function as well as technical applications. Determination of these low-content proteinaceous matters requires a highly sensitive and selective method. In this study, a methodological approach for investigations of the presence of proteinaceous material over the molar mass distribution (*MD*) of polysaccharides was developed using gum acacia (GA) as a model polysaccharide. EDC fluorescence-labeling method was modified by changing the pH (7, 9, and 11) of the solution for the analysis of low-content protein in food materials. Fluorescence spectroscopy and asymmetrical flow field-flow fractionation (AF4) were employed for characterizing the labeling efficiency and physiochemical properties of unlabeled and fluorescence-labeled GA. AF4 provided molar mass (*M*) and the radius of gyration (*r*_*G*_) of arabinogalactan (AG) and arabinogalactan protein complex (AGP) and determined the presence of proteinaceous matter over the *MD*. The labeling efficiencies of GA at pH 7, 9, and 11 determined by fluorescence spectroscopy were 56.5, 68.4, and 72.0%, respectively, with an increment of 15.5% when pH was increased from 7 to 11. The modified EDC fluorescence-labeling method allows highly sensitive and selective analysis of low-content proteinaceous matters and their distribution in natural polysaccharides.

**Graphical abstract:**

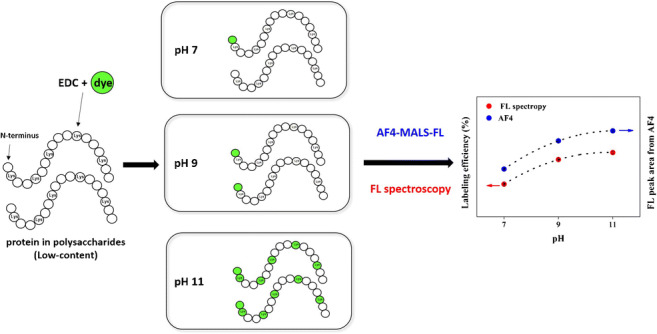

**Supplementary Information:**

The online version contains supplementary material available at 10.1007/s00216-021-03587-y.

## Introduction

Precolumn fluorescent labeling of protein is often used to increase the sensitivity and selectivity of their detection [1–4]. Primary amine (-NH_2_) and cysteine on the proteins can be targeted by selecting appropriate fluorescent dyes and crosslinkers. Among the various crosslinkers, 1-ethyl-3-(3-dimethylaminopropyl) carbodiimide (EDC) is widely used as a water-soluble crosslinker in the field of biochemistry for fluorescence assay of amines, proteins, and carboxylic acids [5–9]. The excessive fluorescent dyes and EDC can be easily removed using dialysis or gel filtration. The reaction mechanism of EDC crosslinking includes two steps. Firstly, EDC activates the carboxyl group by forming an intermediate ester (O-acylisourea) with it (step 1). Secondly, the intermediate further reacts with a primary amine to yield amide bonds (step 2). The EDC crosslinking reaction must be processed rapidly as the intermediate ester can be rapidly hydrolyzed and produce nonreactive hydrolysates in aqueous solutions. The hydrolysis reaction is known to be greatly affected by pH [10]. Acidic and neutral pH has been used to facilitate step 1, and the labeling efficiency can be increased by increasing the EDC content or prolonging the reaction time [11, 12]. However, these strategies are limited, especially for samples with very low protein content, and the prolonged reaction time hinders the analysis of a large number of samples. It is necessary to increase the labeling efficiency furthermore and shorten the reaction time for sensitive and fast analysis of very low-content proteins in samples. It is reported that higher pH is conducive to the reaction of the intermediate and primary amine (step 2) [10, 13–15]. As far as we know, there is no report about fluorescence labeling of protein using the EDC method in a basic environment. It is assumed that higher labeling efficiency and a faster reaction rate can be obtained at high pH in step 2.

Natural polysaccharides often contain a low amount of proteinaceous matter. For example, gum acacia (GA), a type of exudate gum produced by acacia trees when subjected to stresses such as heat, drought, or wounding [16], is widely used as an emulsifier in the food and softdrink industries [17–20]. Studies have shown that GA is a type of heterogeneous polysaccharide, containing about 2% of protein [16, 21–23]. Although the content is low, the proteinaceous matter renders macromolecules surface-active and is responsible for GA’s emulsifying properties. It is reported that the emulsifying capacity of GA is reduced when its protein components are eliminated [24]. The proteinaceous matter in GA is not evenly distributed over the populations. For example, arabinogalactan (AG) and arabinogalactan protein complex (AGP) represent about 90% and 10% of the weight of GA, respectively [25, 26].

In addition, studies have shown that AGP plays a key role in the emulsifying property of GA by giving a pronounced surface activity [27]. It can be related to the structural flexibility and high protein content of AGP than AG [28]. Therefore, the determination of the protein and its distribution over the molar mass distribution (*MD*) is important for understanding the mechanism of emulsification property of polysaccharides such as GA and providing support for developing high-efficiency substitute emulsifiers. Intrinsic fluorescence of natural polysaccharides complex is typically low given that the proteinaceous matters are low in concentration and may not contain fluorescent amino acids in some cases. Hence, fluorescence labeling is necessary for their analysis.

Asymmetrical flow field-flow fractionation (AF4) is a technique with a broad separation range in size and gentle conditions, making it well-suited for polydisperse polysaccharides [29–33]. Pre-separation fluorescence labeling followed by AF4 separation and fluorescence detection has been used to characterize protein in polysaccharides such as GA, starch, *β*-glucan, and mesquite gum [28, 34, 35]. Although compared with UV detection, the fluorescence signal after labeling was improved, in the case of polysaccharides with very low protein content, the intensity of the fluorescence response is still not enough for quantification and further analysis.

In this study, the aim is to develop a methodological approach for investigations of the presence of low-content proteinaceous material over the size and *MD* of polysaccharides. The methodology was developed using GA as a relevant model polysaccharide. To increase the labeling efficiency, and reduce the time needed to accomplish the labeling reaction, one strategy is to increase the number of reactive sites, i.e., primary amines in the sample. Since protonation and deprotonation reactions occur to amino groups when pH is lower or higher than their *pK*_*a*_, the amount of reactive primary amine group for EDC crosslinking reaction should be changed with the pH of the solution. If the pH is increased so that it exceeds the *pK*_*a*_ of most of the primary amine on the N-terminus and the side chains of some amino acids (such as lysine), it could help increase the number of reactive sites, thereby increasing the labeling efficiency. Thus, the effect of pH on fluorescence-labeling efficiency of proteinaceous matter in GA using 7-methoxycoumarin-3-carboxylic acid (MC) as a fluorescent dye and EDC as crosslinker is investigated here. The fluorescent-labeled GA was analyzed using fluorescence spectrometry and AF4 coupled with multiple detectors such as multi-angle light scattering (MALS), differential refractive index detector (dRI), and fluorescence detector (FL).

## Materials and methods

### Materials

The GA sample was obtained from C.E. Roeper GmbH (Hamburg, Germany). 1-Ethyl-3-(3-dimethylaminopropyl) carbodiimide (EDC) and 7-methoxycoumarin-3-carboxylic acid (MC) were purchased from Invitrogen (Lidingö, Sweden), and were used for fluorescence-labeling of the proteinaceous material in GA and bovine serum albumin (BSA). The BSA was purchased from Sigma-Aldrich (St. Louis, MO, USA). The dimethyl sulfoxide (DMSO) was purchased from Sigma-Aldrich (Darmstadt, Germany). Di-sodium hydrogen phosphate dodecahydrate (Na_2_HPO_4_·12H_2_O) and sodium dihydrogen phosphate monohydrate (NaH_2_PO_4_·H_2_O) were purchased from Merck KGaA (Darmstadt, Germany), and were used for the preparation of 10 mM phosphate buffer at pH 7, 9, and 11. Sodium nitrate (NaNO_3_) and sodium azide (NaN_3_) were obtained from Merck KGaA and were used for the preparation of carrier liquid for AF4 analyses. All aqueous solutions were prepared in Milli-Q water (18.2 MΩ/cm) produced by a Milli-Q plus purification system from Millipore Co. Ltd. (Billerica, MA, USA).

### Determination of total protein content of GA

The total protein content was determined by measuring nitrogen content using an elemental analyzer (Flash EA 1112N, Thermo Fisher Scientific, Delft, Netherlands). A small amount (20 to 30 mg) of the sample was weighed and packaged with aluminum foil. The sample package combusted by heating up to 1000 °C in a sealed furnace and the nitrogen content was determined by thermal conductivity detection. The protein content was calculated by a nitrogen-to-protein conversion factor of 6.25.

### Fluorescence labeling with EDC

The GA solution for labeling was prepared at the concentration of 20 mg/mL in 10 mM phosphate buffer at three different pH of 7, 9, and 11. Labeling of protein in GA was accomplished by following the method described by Zielke et al. [35] with minor modification in that 1 mM MC solution was prepared by dissolving it in DMSO and diluting the solution with water 4 times the volume of DMSO followed by 5-min vortexing. Then, the solution was mixed with 1 mM EDC in the volume ratio of 1:1 followed by 3-min vortexing (step 1). Subsequently, the labeling solution was added to the GA solution in the volume ratio of 1:1 and 1 min of vortexing (step 2). After vortexing, the solution was left at room temperature for reaction during 10 min, 1.5 h, 3 h, and 4.5 h, respectively. The samples were directly injected into the AF4 channel for analysis. The final concentration of GA was 10 mg/mL.

The BSA was labeled with the same procedure as GA except that the concentration of BSA solution was 1 mg/mL.

### Determination of labeling efficiency by fluorescence spectroscopy

The fluorescence spectra of labeled BSA and GA samples were measured for determining labeling efficiency using FluoroMate FS-2 (Scinco Co., Ltd., Seoul, South Korea). The excitation and emission spectra were obtained by scanning through the wavelength ranging from 250 to 380 nm and 360 to 500 nm, respectively, and the scan speed was 10 nm/min at a photomultiplier tube (PMT) voltage of 300. All fluorescence spectroscopic measurements were made at room temperature. The collection and processing of data were carried out using the FluoroMaster Plus software (ver. 4.3, Scinco Co., Ltd., Seoul, South Korea).

The fluorescence-labeled sample contains unbound MC that emits fluorescence at a similar wavelength, which interferes with the analysis of fluorescence-labeled samples (BSA and GA). A desalting column (PD-10, GE Healthcare Bio-Sciences Corp., NJ, USA) was used to eliminate the unbound labeling compounds before determination of the labeling efficiency. The desalting column was first equilibrated with 10 mM phosphate buffer at the same pH as that used to label the samples, then loaded with 2.5 mL of fluorescence-labeled sample and centrifuged at 1000×*g* for 2 min. The collected eluate from the desalting column was analyzed by fluorescence spectroscopy.

The labeling efficiency was determined from the emission intensity measured after desalting divided by the emission intensity measured before desalting, as shown in Eq. (1).

1 $$ Labeling\ efficiency\ \left(\%\right)=\frac{Em. intensity\ after\ desalting}{Em. intensity\ before\ desalting}\times 100 $$

### Characterization of labeled GA by AF4-MALS-dRI-FL

The AF4 system was an Eclipse 3+ system (Wyatt Technology, Dernbach, Germany), coupled online with a MALS detector (DAWN HELEOS II, Wyatt Technology), a dRI detector (Optilab T-rEX, Wyatt Technology), and a FL detector (FP-920, Jasco Corporation, Tokyo, Japan) operating at the excitation and emission wavelengths of 340 and 400 nm, respectively.

The AF4 channel was trapezoidal with a tip-to-tip length of 26.5 cm and the width at the inlet and outlet of 2.2 and 0.6 cm, respectively. The channel was made up of a 350-μm-thick Mylar spacer and a regenerated cellulose (RC) membrane (molecular weight cut-off of 10 kDa, Millipore, Bedford, USA). The actual channel thickness was determined to be 235 μm from the retention time (*t*_*R*_) of BSA by AF4 theory using the FFFHydRad 2.2 software [36, 37]. The AF4 carrier liquid was 10 mM phosphate buffer, pumped into the AF4 channel using an Agilent 1200 HPLC pump equipped with an auto-sampler and an in-line vacuum degasser (Agilent Technologies, Waldbronn, Germany). The channel flow rate was kept constant at 1.0 mL/min, while the cross-flow rate was exponentially decreased from 3.0 to 0.1 mL/min with a half-life time of 4 min and then kept constant at 0.1 mL/min for 35 min. The channel was washed with the carrier liquid for 15 min without cross-flow at the end of each run. All AF4 experiments were performed at room temperature. The collection and processing of AF4 data were using the ASTRA ver. 6.1.17 software (Wyatt Technology) with the *d*_n_/*d*_c_ value of 0.141 mL/g for all GA samples [28, 38, 39]. In all cases, the Berry method was used to fit the light scattering data [40, 41].

## Results and discussion

### Verification of improved labeling efficiency by BSA

The effect of pH on labeling efficiency was first tested using BSA. As shown in Table 1, as pH increases from 7 to 11, the labeling efficiency of BSA continuously increased from 55.0 to 87.2%. Proteins have primary amine groups located at the end of peptides (N-terminus). In addition, some amino acids (i.e., asparagine, glutamine, lysine, and arginine) have primary amines on their side chains when the pH of the environment are lower than their *pK*_*a*_. The labeling reaction occurs only with primary amines. At low pH, most primary amines exist in the form of$-{NH}_3^{+}$, which has limited activity for labeling reaction. When pH is higher than their *pK*_*a*_, the primary amines will be deprotonated, and exist in the form of −*NH*_2_, which is reactive for labeling reaction. The *pK*_*a*_ of primary amines on side chains of asparagine, glutamine, lysine, and arginine are 3.86, 4.25, 10.5, and 12.5, respectively, while *pK*_*a*_ of N-terminuses is between 9 and 10. As a result, when the pH of the buffer increases from 7 to 9, some N-terminuses will convert from$-{NH}_3^{+}$ to −*NH*_2_, thus increasing the labeling efficiency. When pH increases from 9 to 11, the $-{NH}_3^{+}$ on the side chains of lysine convert to −*NH*_2_, further increasing the labeling efficiency. According to Table 1, at pH 11, the increase in labeling efficiency compared with pH 9 is greater than the content of lysine in BSA, which is about 12% as reported [42]. This excessive increment may be attributed to two possibilities. Firstly, some N-terminuses remained as $-{NH}_3^{+}$ at pH 9 because they have *pK*_*a*_ range of 9 to 10, thus more converting to −*NH*_2_ at pH 11 (i.e., more dominant form of N-terminuses is −*NH*_2_ at pH 11 than pH 9). Secondly, BSA underwent structural changes with pH, which means the energy level of BSA was changed, resulting in a change the fluorescence emission intensity [43, 44].

**Table 1:** The labeling efficiency of BSA at pH 7, 9, and 11

pH of solution	**Labeling efficiency (%)**
7	55.0
9	63.3
11	87.2

### Analysis of labeled GA by fluorescence spectroscopy

The total protein content of GA was measured to be 1.87 ± 0.13 wt% (Table S1). The effect of pH on the labeling efficiency of GA was then measured by fluorescence spectroscopy. Firstly, the fluorescence spectra of GA and labeling solution (mixture of MC and EDC) were examined respectively. Fig. S1 shows the excitation and emission spectra GA solutions and prepared at three different pH values (7, 9, and 11) and the labeling solution. It was found that GA does not give rise to any fluorescence emission at the excitation wavelength of 373 nm, while the labeling solution shows the maximum emission wavelength of 410 nm at the excitation wavelength of 373 nm. Fig. S2 shows the fluorescence spectra of GA samples labeled at three different pH. After the labeling of GA, the maximum excitation and emission wavelengths of MC were shifted from 373 to 340 and 410 to 400 nm, respectively. This blue shift is presumably due to a change in the chemical structure of the labeling compound when it binds to GA, causing changes in the quantum energy levels. In addition, it is unable to monitor the difference in labeling efficiency between pH values because all labeled samples include unbound MC. Therefore, a desalting column was used to remove the unbound MC in the labeled samples. Fig. S3 showed that the removal efficiency of excessive reagents was >90% after one pass and >99% after two passes through the desalting column, respectively. Two-pass desalting was used for all labeling efficiency studies.

**Fig. 1:**
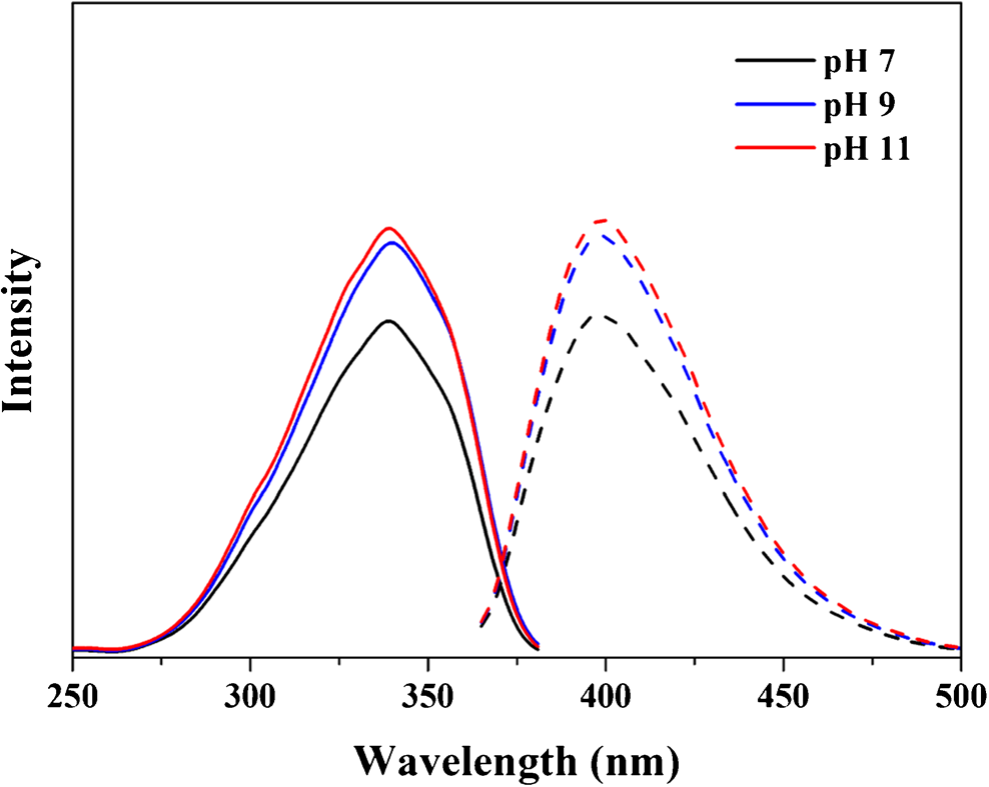
Fluorescence spectra of GA labeled at pH 7 (black), 9 (blue), and 11 (red) after desalting. The solid and dashed lines represent excitation and emissions spectra, respectively

Figure 1 shows the fluorescence spectra of GA labeled at pH of 7, 9, and 11 obtained after desalting, where a significant increase in the intensity of both excitation (solid lines) and emission (dashed lines) responses with the increase of pH can be observed. Table 2 shows the labeling efficiencies, calculated by Eq. (1). The improvement in labeling efficiency of GA with increasing pH agrees well with the results of BSA, which is mainly caused by the change of N-terminus when pH is increased from 7 to 9, and by that of the primary amine on the side chain of lysine when pH is increased from 9 to 11. It is worth noting that the labeling efficiency at pH 11 increased by 3.6% compared with pH 9, which is comparable with the content of lysine in the amino acid composition of GA (3.1%) (Table S2).

**Table 2:** The labeling efficiency of GA at pH 7, 9, and 11

**pH**	**Emissions intensity at 400 nm (×10**^**4**^**)**		**Labeling efficiency** **(%)**
	**Before desalting**	**After desalting**	
**7**	1.86 ± 0.02	1.05 ± 0.02	56.5 ± 0.5
**9**	1.87 ± 0.02	1.28 ± 0.01	68.4 ± 0.3
**11**	1.86 ± 0.01	1.34 ± 0.01	72.0 ± 0.5

### Analysis of labeled GA by AF4-MALS-dRI-FL

**Fig. 2:**
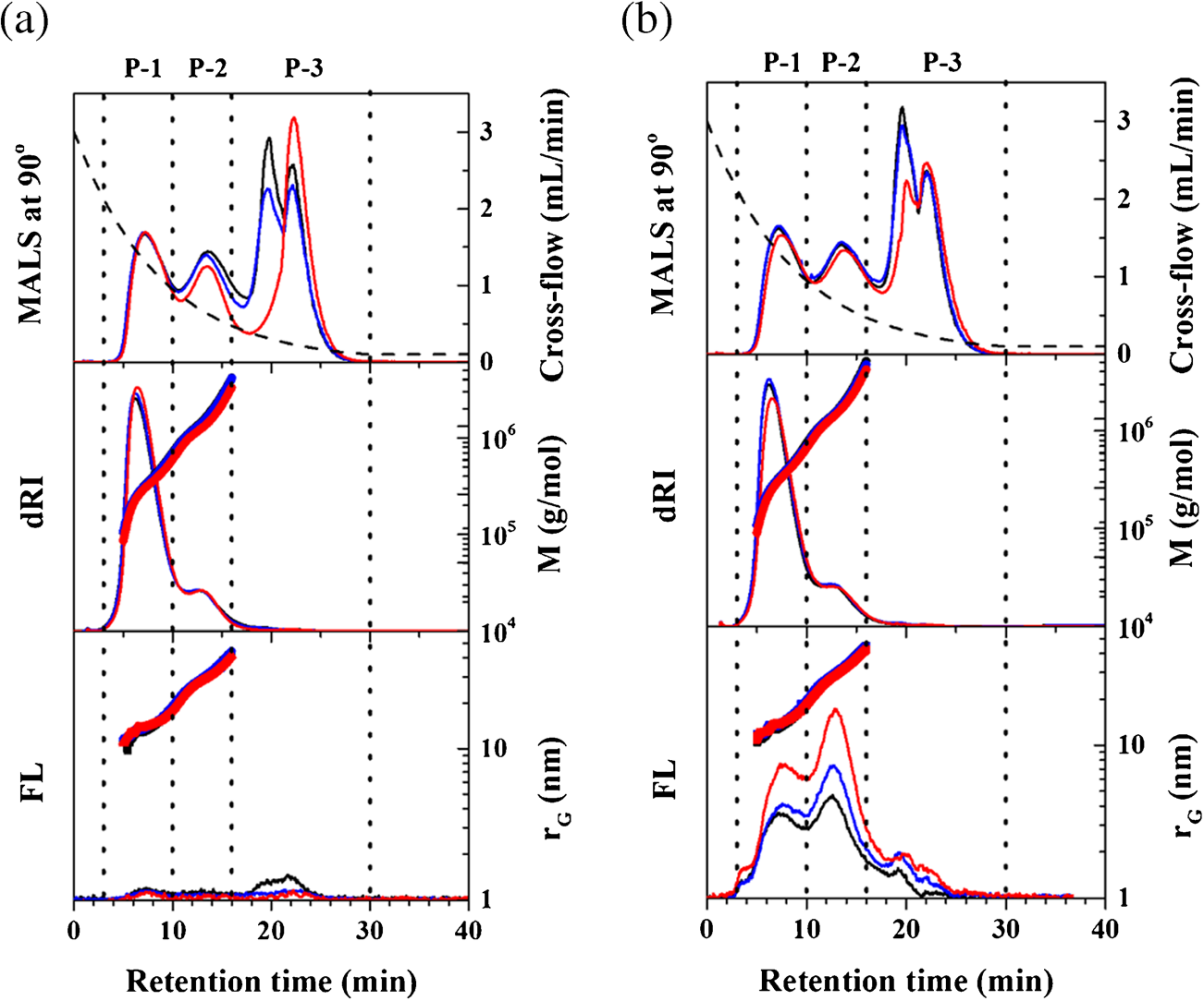
AF4 fractograms obtained from MALS_90_, dRI, and FL detectors of unlabeled (**a**) and labeled GA measured after 10 min of labeling reaction (**b**) at pH 7 (black), 9 (blue), and 11 (red line). The right y-axes of both (**a**) and (**b**) represent cross-flow rate (dashed line), *M* (scatter), and *r*_*G*_ (scatter) from top to bottom. P-1, P-2, and P-3 are population eluted at 3–10, 10–16 min, and 16–30 min, respectively. The *M* and *r*_*G*_ of P-3 were not determined due to the low dRI signal

Figure 2 shows the AF4 fractograms of unlabeled (Fig. 2a) and labeled (Fig. 2b) GA at pH of 7, 9, and 11. For each of them, AF4 fractograms obtained from MALS_90_ (MALS signal measured at the scattering angle of 90°), dRI, and FL detectors are shown from top to bottom. In all AF4 analyses, the cross-flow rate was programmed (exponential decay, dashed line) as shown in the MALS_90_ fractograms. The *M* and *r*_*G*_ were determined for each slice of the fractograms and is shown with the dRI fractograms and the FL fractograms for both samples.

The MALS_90_ fractograms show there are three major populations in both unlabeled and labeled GA (P-1, P-2, and P-3), eluting at *t*_*R*_ range of 3 to 10, 10 to 16, and 16 to 30 min, respectively. The P-1 and P-2 of *M* range 1.0×10^5^ to 9.0×10^5^ and 9.0×10^5^ to 5.0×10^6^ g/mol, respectively. The *M* of P-3 was not determined because the dRI signals were too weak.

The *M* and *r*_*G*_ determined for the GA samples shown in Fig. 2 are summarized in Table 3. The *M* and *r*_*G*_ in Table 3 are in good agreement with those reported in literature where GA was analyzed by size exclusion chromatography (SEC) or AF4 coupled with MALS [28, 45], according to which the P-1 and P-2 correspond to arabinogalactan (AG) and arabinogalactan protein (AGP), respectively [28, 38, 45, 46]. The P-3 is likely to be composed of aggregates that could be formed by the self-assembly of AGP and is known to be present in plant secretions containing AGP [32].

**Table 3:** The *M* and *r*_*G*_ of P-1 and P-2 of unlabeled and fluorescence-labeled GA at pH 7, 9, and 11

**Type**	**pH**	**P-1 (AG)**		**P-2 (AGP)**	
		***M*** **(g/mol)**	*r*_*G*_ **(nm)**	***M*** **(g/mol)**	*r*_*G*_ **(nm)**
**Unlabeled**	**7**	3.5 × 10^5^ (± 0.8%)	14 (± 13.3%)	1.7 × 10^6^ (± 0.6%)	30 (± 2.2%)
	**9**	3.5 × 10^5^ (± 0.8%)	15 (± 13.0%)	1.6 × 10^6^ (± 0.8%)	31 (± 3.0%)
	**11**	3.2 × 10^5^ (± 0.8%)	15 (± 16.6%)	1.4 × 10^6^ (± 1.1%)	29 (± 4.3%)
**Labeled**	**7**	3.6 × 10^5^ (± 0.8%)	14 (± 13.7%)	1.9 × 10^6^ (± 0.8%)	31 (± 2.9%)
	**9**	3.6 × 10^5^ (± 0.9%)	15 (± 13.0%)	1.8 × 10^6^ (± 0.9%)	31 (± 2.9%)
	**11**	3.5 × 10^5^ (± 0.9%)	15 (± 13.4%)	1.7 × 10^6^ (± 0.9%)	30 (± 3.3%)

In Table 3, the *M* and *r*_*G*_ of GA show a slight decrease or increase depending on the conditions (pH values and before/after labeling). However, it is difficult to explain this change by binding of MC on GA because the same dn/dc value was used for all GA samples, although its value may change slightly under different circumstances.

As shown in the FL fractograms in Fig. 2, the FL intensities of the labeled GA are significantly enhanced compared with those of the unlabeled GA. As predicted, the fluorescence intensity increases with pH. P-2 shows higher responses than P-1, even though the concentration of P-2 is substantially lower than P-1, as indicated by the dRI responses. It can be inferred that P-2 contains more proteinaceous matter than P-1, which agrees with previous literature [16, 22, 28, 33, 45, 47, 48].

**Fig. 3:**
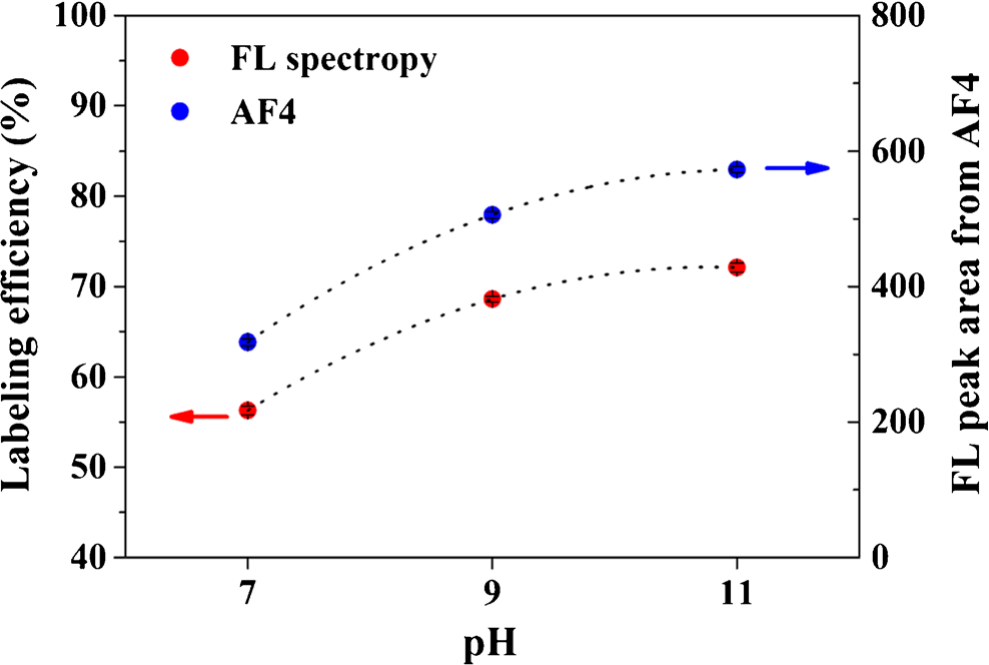
Labeling efficiencies obtained from maximum emission intensity of fluorescence spectrometer (left, red) and peak area of AF4-FL fractogram (right, blue)

Figure 3 shows the labeling efficiencies measured from the maximum emission intensity in fluorescence spectroscopy obtained from offline fluorometers (left y-axis) and the peak area of the FL fractograms obtained from fluorescence detector coupled online with AF4 (right y-axis). Both of them increase similarly as pH increases from 7 to 11, indicating that more primary amines are combined with fluorescent reagents at higher pH. As a result, the sensitivity of AF4 measurement is greatly enhanced at pH 11. In addition, the labeling reaction is also accelerated at higher pH. Figure S4 shows the normalized peak area of the AF4 fractogram obtained from the FL response. The normalized peak area obtained by injection immediately after mixing GA with labeling solution at pH 11 is close to 90%, much higher than that at pH 7 and 9.

**Fig. 4:**
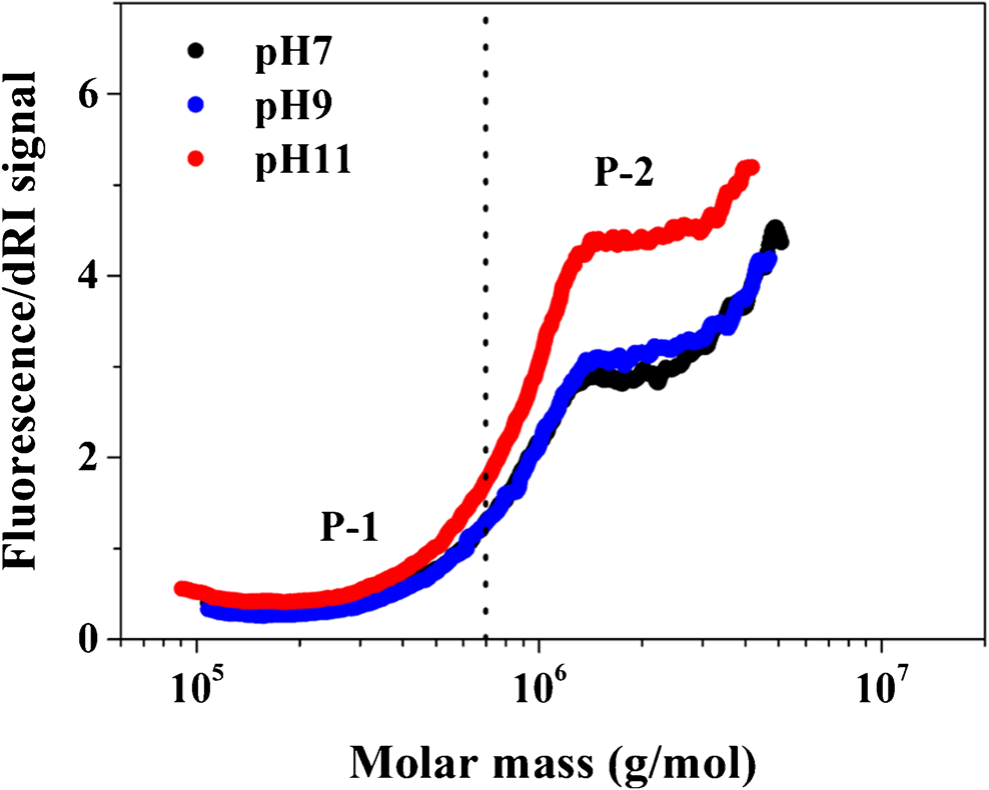
Fluorescence/dRI ratio vs. *M* plot of GA at pH 7 (black), 9 (blue), and 11 (red). The dotted line divides P-1 and P-2

The ratio of fluorescence response to dRI response gives a relative estimation of the protein content. Figure 4 shows FL/dRI ratio vs. *M* plot of P-1 and P-2 of GA at pH 7, 9, and 11, indicating the distribution of proteinaceous matter over the *MD*. The results show that P-2 is richer in protein than P-1; thus, the increased labeling efficiency is more obviously displayed by P-2.

## Conclusion

In summary, this study modified the widely used EDC fluorescence-labeling method for determining the distribution of low-content protein in natural polysaccharides. As a representative sample, the influence of pH (7, 9, and 11) on the labeling efficiency of GA was evaluated by batch FL and coupling FL detector online with AF4-MALS-dRI. The results showed that the labeling efficiency was greatly improved by increasing pH. According to FL results, the labeling efficiencies at 7, 9, and 11 were 56.5, 68.4, and 72.0%, respectively. AF4 results also showed a consistent increase in the FL response with pH. The distribution of protein over *MD* was evaluated by FL/dRI vs. *M* plot (see Fig. 4) and showed that AGP is more protein-rich than AG. Furthermore, the labeling reaction rate was greatly improved at high pH. The improved labeling efficiency indicated that more primary amines combined with the fluorescent reagent because of deprotonation of the primary amines on the side chain of lysine and the N-terminus when pH was higher than their *pK*_*a*_. The method and results presented in this study should help determine the distribution of low-content proteinaceous matters over the whole *M* range in polysaccharides.

## Supplementary Information

ESM 1 (PDF 497 kb)
