# Supplementary material for: Modification of EDC method for increased labeling efficiency and characterization of low-content protein in gum acacia using asymmetrical flow field-flow fractionation coupled with multiple detectors
Source: Anal Bioanal Chem. 2021 Aug 20;413(25):6313–20. doi: 10.1007/s00216-021-03587-y (PMC8487880; doi:10.1007/s00216-021-03587-y)
Supplement: Supplementary file 1 — (PDF 497 kb) [file 216_2021_3587_MOESM1_ESM.pdf]

Supplementary information

Modification of EDC method for increased labeling efficiency and characterization of low-content protein in Gum Acacia using asymmetrical flow field-flow fractionation coupled with multiple detectors

Meiyu Zhang[1], Lars Nilsson[2,*], Seungho Lee[1,*], Jaeyeong Choi[1,*]

[1]*Department of Chemistry, Hannam University, Daejeon* 34430, *South Korea*

[2]*Department of Food Technology, Engineering and Nutrition, LTH, Lund University, Lund, Sweden*

*** Co-corresponding authors at:**

Lars Nilsson: *lars.nilsson@food.lth.se*

Seungho Lee: *slee@hnu.kr*

Jaeyeong Choi: *feelcjy@gmail.com*

Table S1 Total protein content of Gum Acacia (GA) determined by nitrogen content

No.	Protein content (%)
1	2.01
2	1.84
3	1.75
Average	1.87 ± 0.13

Table S2 Amino acid contents in gum Arabic from Acacia Senegal (Mahendran et al., 2008) and pKa values for N-terminal and side chain of amino acids (The Merck Index)

Amino acid (AA)	% Amino acid in GA	NH_2 in side chain	pKa	
			α-NH_3^+	side chain
Hydroxyproline	0.711	-	9.7	-
Serine	0.302	-	9.2	-
Threonine	0.208	-	9.1	-
Proline	0.180	-	10.6	-
Leucine	0.198	-	9.6	-
Histidine	0.166	-	9.2	6.0
Aspartic acid	0.141	-	9.8	3.9
Glutamic acid	0.122	-	9.7	4.3
Valine	0.085	-	9.6	-
Phenylalanine	0.105	-	9.1	-
Lysine	0.075	O	9.0	10.5
Alanine	0.045	-	9.7	-
Isoleucine	0.031	-	9.7	-
Tyrosine	0.042	-	9.1	10.8
Arginine	0.037	O	9.0	12.5
Methionine	0.002	-	9.2	-
Cysteine	0.000	-	10.8	8.3
Tryptophan	0.000	-	9.4	-
Total amino acids	2.450	-	-	-
The proportion of lysine in amino acid composition	3.06			

(a)

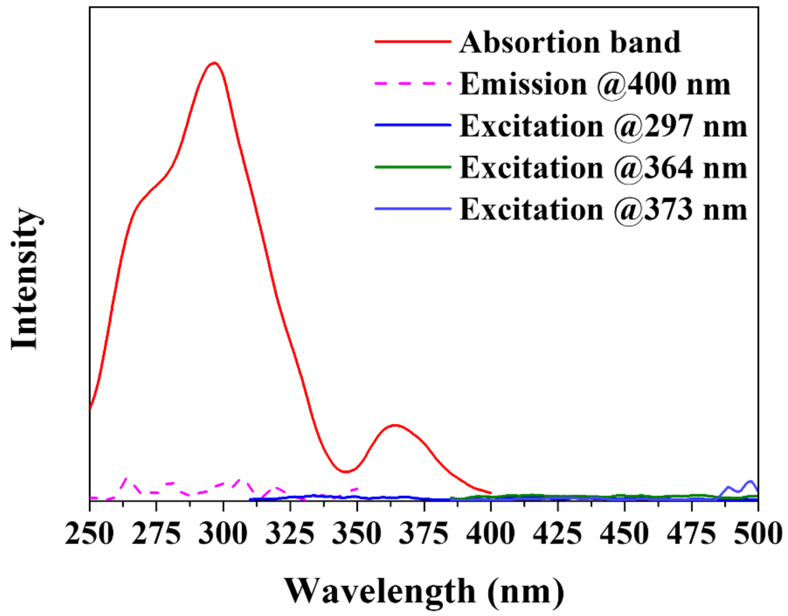
Absorption band
Emission @400 nm
Excitation @297 nm
Excitation @364 nm
Excitation @373 nm
Intensity
250 275 300 325 350 375 400 425 450 475 500
Wavelength (nm)

(b)

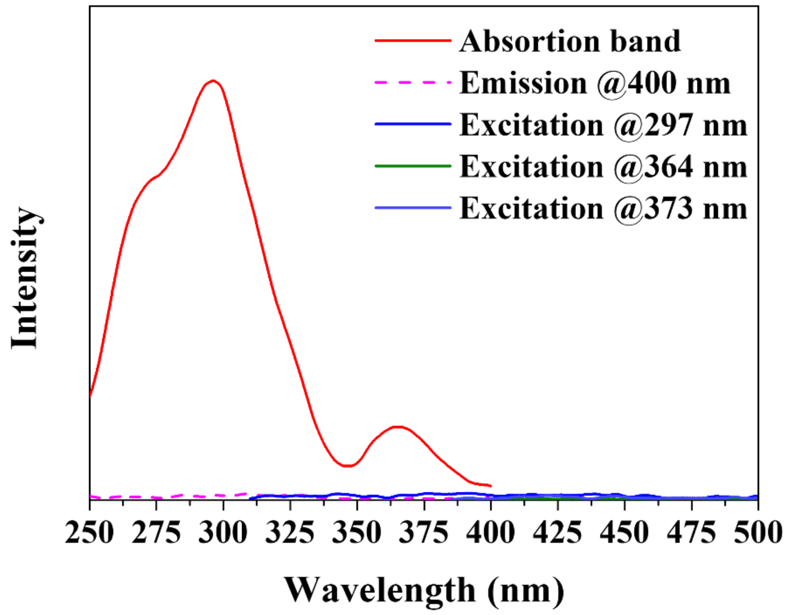
Absorption band
Emission @400 nm
Excitation @297 nm
Excitation @364 nm
Excitation @373 nm
Intensity
250 275 300 325 350 375 400 425 450 475 500
Wavelength (nm)

(c)

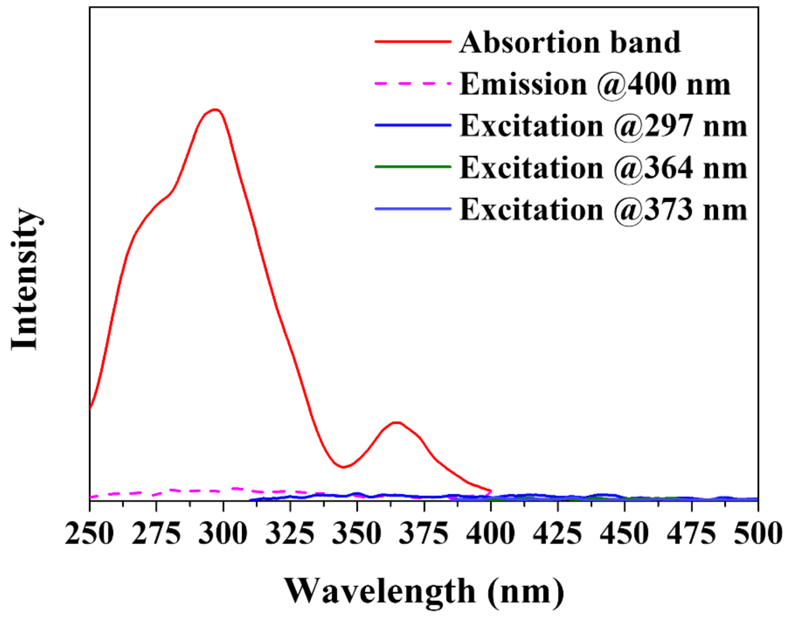

(d)

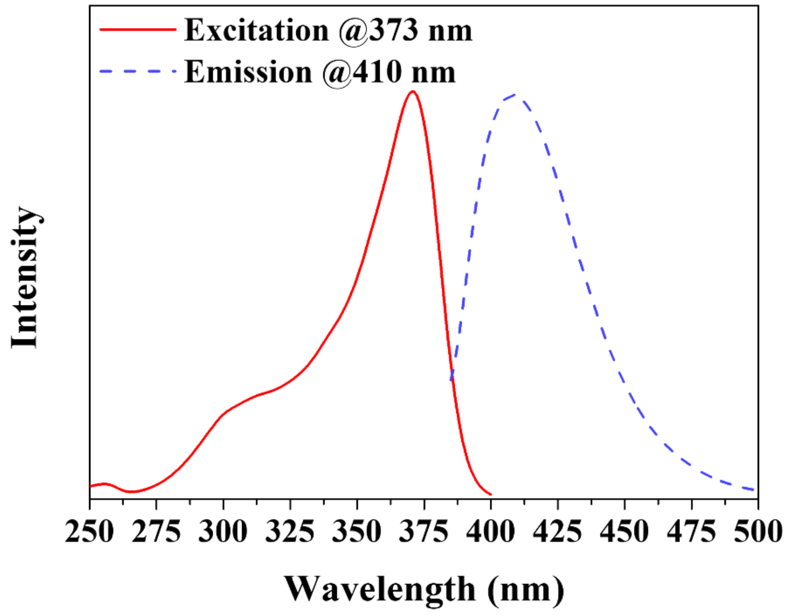

Fig. S1 Fluorescence spectra of GA solutions at three different pH values and labeling solution. (a) GA at pH 7, (b) GA at pH 9, (c) GA at pH 11, and (d) labeling solution at pH 7. The solid lines and dashed lines represent excitation and emissions spectra, respectively

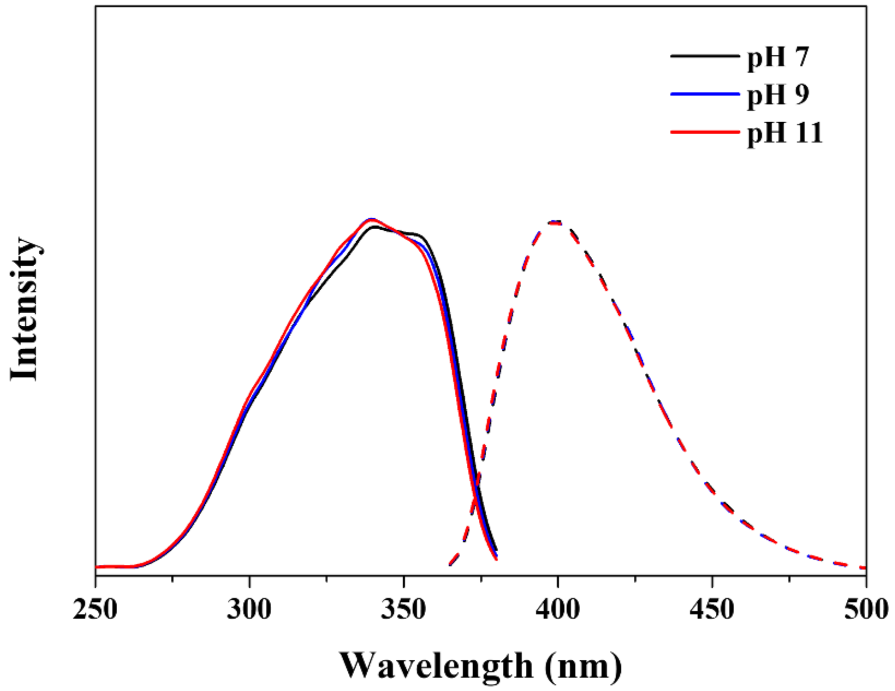

Fig. S2 Fluorescence spectra of labeled GA samples at pH of 7 (black), 9 (blue), and 11 (red) before desalting. The solid lines and dashed lines represent excitation and emission spectra, respectively

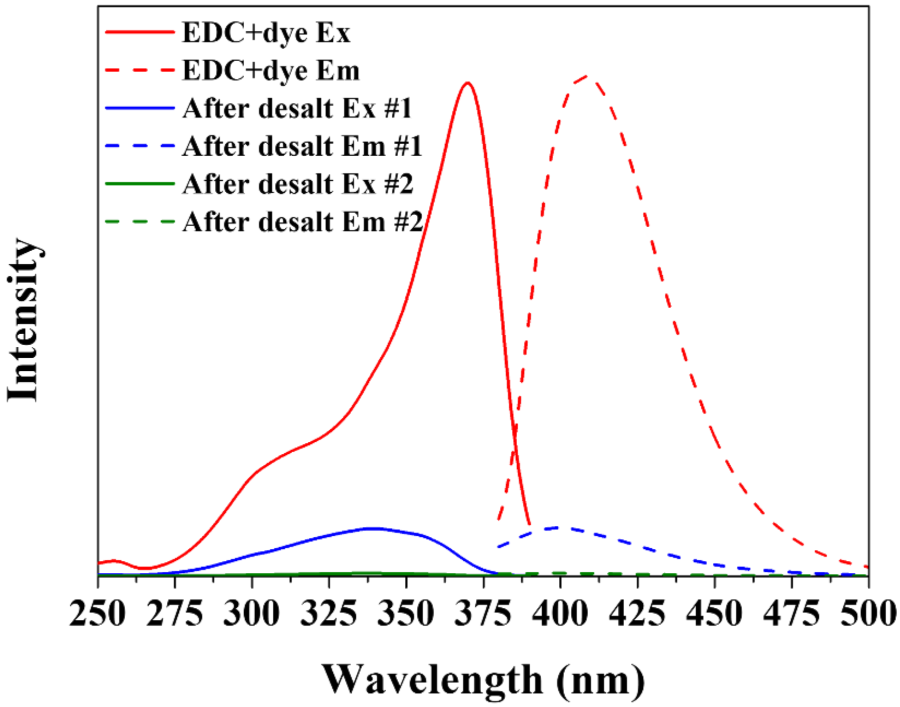

Fig. S3 Fluorescence spectra of labeling solutions before desalting (red), after once (blue) and twice (green) of desalting. The solid lines and dashed lines represent excitation and emission spectra, respectively

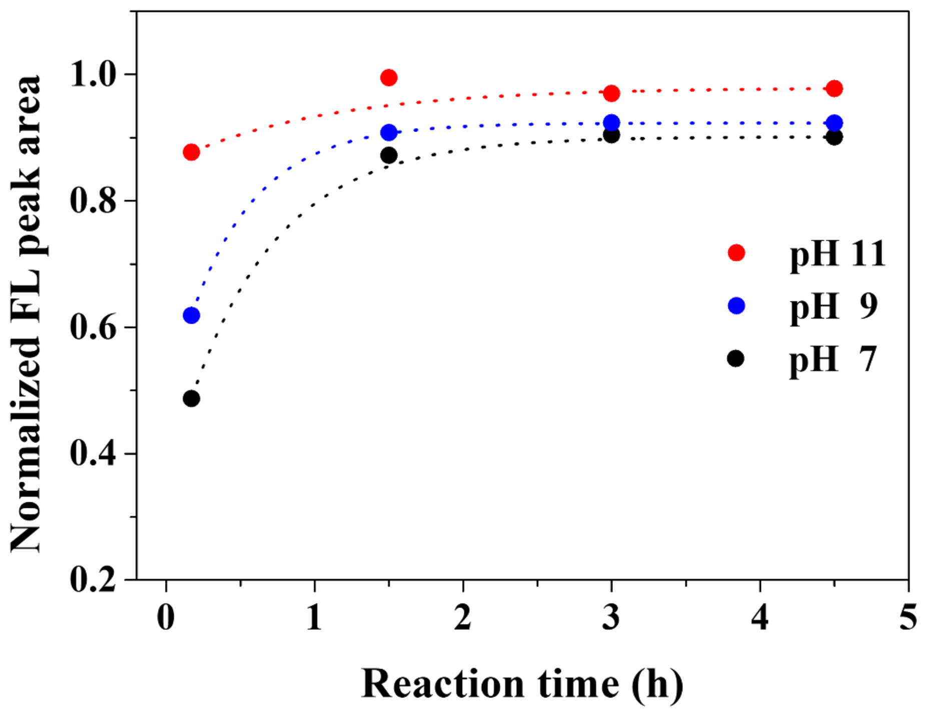

Fig. S4 Normalized AF4-FL peak area of GA labeled at pH 7 (black), 9 (blue), 11 (red) with varied reaction times (10 min, 1.5 h, 3h, and 4.5h)
